# Optimization of Tabersonine Methoxylation to Increase Vindoline Precursor Synthesis in Yeast Cell Factories

**DOI:** 10.3390/molecules26123596

**Published:** 2021-06-11

**Authors:** Pamela Lemos Cruz, Natalja Kulagina, Grégory Guirimand, Johan-Owen De Craene, Sébastien Besseau, Arnaud Lanoue, Audrey Oudin, Nathalie Giglioli-Guivarc’h, Nicolas Papon, Marc Clastre, Vincent Courdavault

**Affiliations:** 1 EA2106 “Biomolécules et Biotechnologies Végétales”, Université de Tours, 37000 Tours, France; pamela.lemos@univ-tours.fr (P.L.C.); natalja.kulagina@univ-tours.fr (N.K.); gregory.guirimand@univ-tours.fr (G.G.); johan-owen.decraene@univ-tours.fr (J.-O.D.C.); sebastien.besseau@univ-tours.fr (S.B.); arnaud.lanoue@univ-tours.fr (A.L.); audrey.oudin@univ-tours.fr (A.O.); nathalie.guivarch@univ-tours.fr (N.G.-G.); marc.clastre@univ-tours.fr (M.C.); 2 Graduate School of Sciences, Technology and Innovation, Kobe University, Kobe 657-8501, Japan; 3 Le Studium Loire Valley Institute for Advanced Studies, 45000 Orléans & Tours, France; 4 Univ Angers, Univ Brest, GEIHP, SFR ICAT, F-49000 Angers, France; nicolas.papon@univ-angers.fr

**Keywords:** alkaloids, *Catharanthus roseus*, metabolic engineering, O-methyltransferase

## Abstract

Plant specialized metabolites are widely used in the pharmaceutical industry, including the monoterpene indole alkaloids (MIAs) vinblastine and vincristine, which both display anticancer activity. Both compounds can be obtained through the chemical condensation of their precursors vindoline and catharanthine extracted from leaves of the Madagascar periwinkle. However, the extensive use of these molecules in chemotherapy increases precursor demand and results in recurrent shortages, explaining why the development of alternative production approaches, such microbial cell factories, is mandatory. In this context, the precursor-directed biosynthesis of vindoline from tabersonine in yeast-expressing heterologous biosynthetic genes is of particular interest but has not reached high production scales to date. To circumvent production bottlenecks, the metabolic flux was channeled towards the MIA of interest by modulating the copy number of the first two genes of the vindoline biosynthetic pathway, namely tabersonine 16-hydroxylase and tabersonine-16-*O*-methyltransferase. Increasing gene copies resulted in an optimized methoxylation of tabersonine and overcame the competition for tabersonine access with the third enzyme of the pathway, tabersonine 3-oxygenase, which exhibits a high substrate promiscuity. Through this approach, we successfully created a yeast strain that produces the fourth biosynthetic intermediate of vindoline without accumulation of other intermediates or undesired side-products. This optimization will probably pave the way towards the future development of yeast cell factories to produce vindoline at an industrial scale.

## 1. Introduction

Plant specialized metabolites were first described in the middle 1920s and were initially named secondary metabolites because these compounds were thought to be non-essential for plant development, in contrast to primary metabolites. Now, accumulating evidence has established that specialized metabolites allow plants to cope with their environment and notably contribute to the arsenal of defenses that plants have evolved against bioaggressors, making specialized metabolites essential components of plant life [1,2,3]. Biological activities of specialized metabolites also explain why they have been associated with medicinal plant properties commonly used in traditional medicine [4,5]. Several of these metabolites, such as the analgesic morphine, the antitussive codeine from *Papaver somniferum* [6], and the antimalarial artemisinin from *Artemisia annua* [7], for instance, have been of great importance to modern medicine for decades. Along with other active plant-born compounds, they constitute around 25% of all the drugs currently used in human health [8].

**Figure 1:**
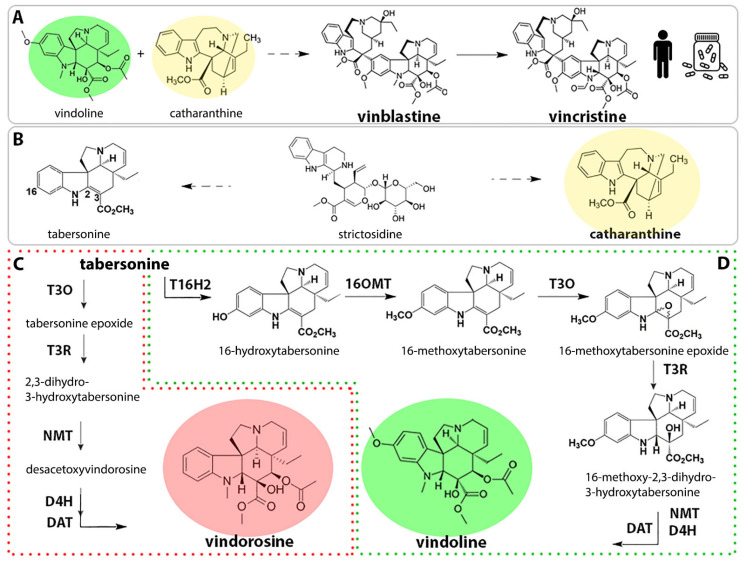
Vinblastine and vincristine production via semi-synthetic synthesis. (**A**) Illustration of the semi-synthetic synthesis of vinblastine and vincristine. (**B**) Tabersonine and catharanthine biosynthesis from strictosidine. (**C**,**D**) Tabersonine bioconversion in planta (**C**): by-product vindorosine biosynthesis. (**D**): vindoline biosynthesis). Solid line: one enzymatic step, discontinuous line: more than one enzymatic step.

Monoterpene indole alkaloids (MIAs) are one of the prominent examples of plant specialized metabolites used in our current pharmacopeia. Almost all MIAs derive from the common precursor strictosidine that is further metabolized to yield the plethora of MIAs accumulated in Apocynaceae, Rubiaceae, Nyssaceae, and Loganiaceae [9]. Among MIAs, vinblastine and vincristine, both specific to the Madagascar periwinkle (*Catharanthus roseus*) [10], are widely exploited in anticancer treatments [11,12,13]. The supply of these compounds mostly relies on plant culture and on the extraction of their two precursors, vindoline and catharanthine, from *C. roseus* leaves, which are further condensed to produce the active compounds (Figure 1A) [14,15]. This supply is obviously highly dependent on plant growth and suffers from recurrent shortages. On the other hand, the chemical synthesis of these molecules is laborious and inapplicable at an industrial scale due to their structural complexity [16,17,18,19], while in vitro cell cultures still fail to produce these MIAs [20].

Alternatively, the recent development of metabolic engineering strategies and heterologous productions provide new perspectives towards the supply of plant molecules of interest [21]. These production strategies basically rely on the reconstitution of a biosynthetic pathway into a heterologous host through gene transfer. Among the potential heterologous hosts, yeast is considered as one of the most suitable organisms for metabolic engineering due to its fast growth, easy genetic manipulation, and available genome sequence [22]. Following the seminal heterologous productions of artemisinin [23], hydrocortisone [24], and progesterone [25], several plant alkaloids have been more recently biosynthesized by recombinant yeast, such as MIAs [26,27,28] but also benzylisoquinoline [29,30,31,32] and tetrahydroisoquinoline [33,34] alkaloids. However, heterologous biosynthesis of MIAs remains challenging due to the high complexity of the pathway and the elaborate cellular and subcellular compartmentalization of enzymes [35,36,37]. For instance, the central MIA precursor strictosidine was de novo produced in yeast at 0.5 mg/L [26], demonstrating the difficulty of reconstituting the entire metabolic pathway and obtaining high-scale production from glucose. By contrast, precursor-directed production, relying on yeast being fed highly abundant biosynthetic intermediates, represents an appealing alternative. Tabersonine is indeed an abundant MIA produced from strictosidine (Figure 1B) and accumulated in the seeds of *Voacanga africana* (25 to 30 g of tabersonine per kg of seed [38]). While tabersonine can be further metabolized into several derivatives, including, for instance, melodinine K [39], this compound is also converted into vindoline in *C. roseus* [40]. As such, tabersonine thus represents a highly valuable compound that can be used to deploy a precursor-directed synthesis of vindoline in engineered yeasts. However, although this bioconversion has been described in yeast [16], only a modest vindoline yield of 1.1 mg·L^−1^ 12 h^−1^ was reached, thus shedding light on the requirement of further optimizations of this strategy.

In *C. roseus* leaves, the tabersonine-to-vindoline conversion involves a biosynthetic route composed of seven steps [16]. Firstly, tabersonine is hydroxylated by tabersonine-16-hydroxylase (T16H2) to produce 16-hydroxytabersonine [41,42,43], followed by an *O*-methylation by tabersonine-16-*O*-methyltransferase (16OMT) [44,45]. The resulting 16-methoxytabersonine is then epoxidized by tabersonine 3-oxygenase (T3O) [46] and reduced by tabersonine 3-reductase (T3R) [16,45], generating the 16-methoxy-2,3-dihydro-3-hydroxytabersonine (Figure 1B). Finally, vindoline is produced through three further reactions of N-methylation [47], hydroxylation, and acetylation [48], catalyzed by 3-hydroxy-16-methoxy-2,3-dihydrotabersonine N-methyltransferase (NMT), desacetoxyvindoline-4-hydroxylase (D4H), and deacetylvindoline 4-*O*-acetyl transferase (DAT) [45,48,49], respectively (Figure 1C). Due to substrate promiscuity, T3O is also able to directly metabolize tabersonine to produce tabersonine epoxide, which is further converted into vindorosine and its biosynthetic intermediates by downstream enzymes T3R, NMT, D4H, and DAT [16,43,46] (Figure 1C).

Since vindorosine cannot be methoxylated a posteriori, vindorosine production affects, in turn, the synthesis of vinblastine and vincristine because it lacks the functional group involved in condensation with catharanthine [43]. Interestingly, a similar hijacking reaction was also observed in the engineered yeast expressing the vindoline pathway [16]. In these conditions, the production of vindorosine even exceeded vindoline synthesis and was accompanied by the massive accumulation of biosynthetic intermediates from both pathways. Therefore, the tight control of the metabolic flux in yeast constitutes a main concern for an optimal production of vindoline through tabersonine bioconversion with reduced accumulation of intermediates and limited vindorosine synthesis. A similar rationale has been already pointed out for benzylisoquinoline [29,30,31,32] and tetrahydroisoquinoline [33,34] alkaloids. 

The present study aimed at optimizing the vindoline production through tabersonine conversion in yeast by resolving the bottleneck of early enzymatic steps to direct the metabolic flux towards vindoline. The first two enzymatic steps of the conversion were thus investigated by analyzing substrate permeability to yeast membranes, enzyme subcellular localization, enzyme isoform, and the effect of gene copy number to prevent tabersonine uptake by the competing T3O. By fine-tuning the copy number of T16H2 and 16OMT, we successfully orientated the conversion of tabersonine towards vindoline and limited biosynthetic intermediate accumulation. In addition, we also drastically prevented the formation of vindorosine precursors, thus offering new perspectives towards the high production of vindoline in yeast cell factories and for other syntheses of natural products in yeast that suffer from endogenous substrate competition.

## 2. Results

### 2.1. Identification of Early Pathway Bottlenecks

To optimize the synthesis of vindoline in engineered yeast and reduce the biosynthetic flux towards the undesired vindorosine (Figure 1C), we first investigated the competition between T16H2 and T3O for tabersonine. Using galactose-inducible episomal vectors of expression, we compared the effect of the T16H2/T3O gene copy ratio on 16-hydroxytabersonine and tabersonine epoxide synthesis (Table 1). 

**Figure 2:**
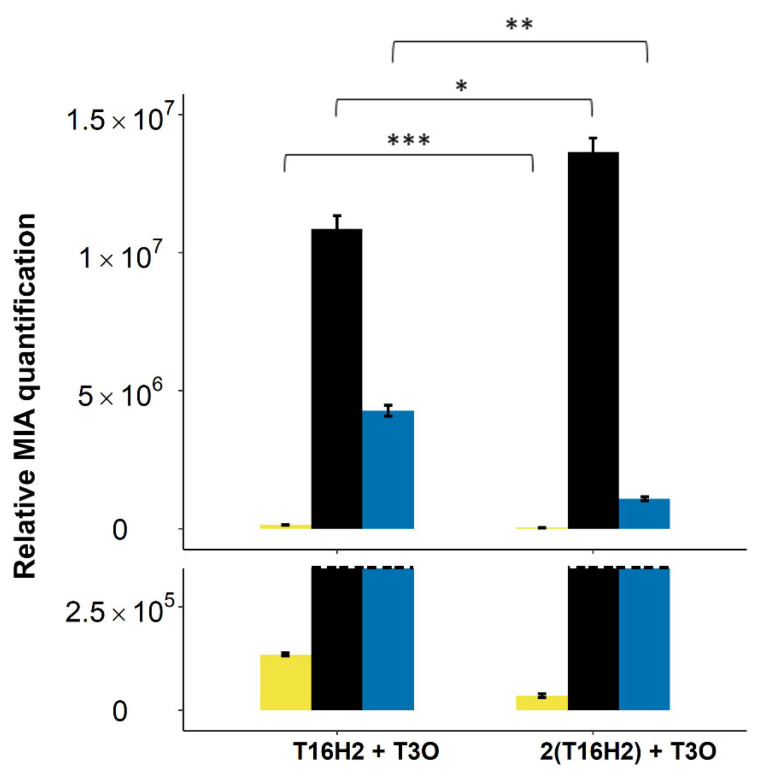
Evaluation of the competition between T16H2 and T3O for tabersonine in yeast. Alkaloids were quantified by UPLC-MS in the yeast culture medium 24 h post-feeding with tabersonine (250 µM). The dashed line represents the scale cut for the visualization of accumulated intermediates of low volume. The yeast strain harbors episomal plasmids containing one or two copies of T16H2 and one copy of T3O. Light yellow = tabersonine, black = 16-hydroxytabersonine, blue = tabersonine epoxide. Statistical analyses were performed with a two-tailed *t*-test (*p* ≤ 0.1, *: *p* ≤ 0.05, **: *p* ≤ 0.01, ***: *p* ≤ 0.001). MIA composition of the yeast culture medium is expressed as relative peak areas.

One or two copies of T16H2 along with one copy of T3O were co-expressed in the *Saccharomyces cerevisiae* Wat11 strain fed with 250 µM of tabersonine. The MIA contents of the extracellular media were analyzed by UPLC-MS 24 h post-feeding (Supplemental Figure S1) since close to 95% of vindoline pathway intermediates are secreted into the culture medium [16]. In the presence of a single T16H2 copy, tabersonine was almost fully consumed (Figure 2). This led to the synthesis of both 16-hydroxytabersonine and tabersonine epoxide in a circa 70/30 ratio, in agreement with the higher affinity of T16H2 for tabersonine [33,36]. Interestingly, the addition of a second T16H2 copy resulted in a significantly increased consumption of tabersonine, accompanied by a 1.25-fold higher production of 16-hydroxytabersonine and a 4-fold decrease in tabersonine epoxide biosynthesis, making 16-hydroxytabersonine the most abundant compound by far (>90%; Figure 2). This demonstrated that increasing the T16H2 gene copy number versus T3O could constitute a promising strategy to reduce vindorosine formation.

**Figure 3:**
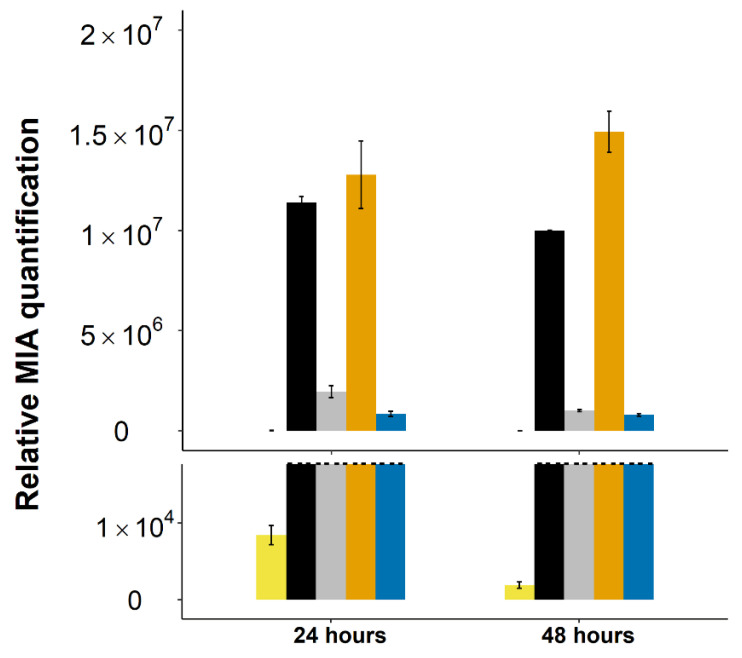
Evolution of MIA biosynthetic intermediates in the medium culture of yeast harboring episomal plasmids containing two copies of T16H2 and one copy of 16OMT and T3O (2(T16H2)_T3O strain). The dashed line represents the scale cut for the visualization of accumulated intermediates of low volume. Alkaloids were quantified by UPLC-MS in the yeast culture medium 24 h post-feeding with tabersonine (250 µM). Light yellow = tabersonine (tab), black = 16-hydroxytabersonine (16OHtab), grey = 16-methoxytabersonine (16MeOtab), dark yellow = 16-methoxytabersonine epoxide (16MeOtab_epoxi), blue = tabersonine epoxide. Error bars correspond to the standard error of biological replicates (*n* = 3). MIA composition of the yeast culture medium is expressed as relative peak areas.

Based on this first observation, we next co-expressed 16OMT from *C. roseus* using the same plasmid system in the yeast strain expressing two copies of T16H2 and one of T3O (Table 1). Twenty-four hours after tabersonine feeding, 16-methoxytabersonine epoxide, which results from the subsequent activity of T16H2/16OMT/T3O, appeared as the main MIA accumulated in the culture medium with low amounts of tabersonine epoxide (Figure 3). However, while low amounts of 16-methoxytabersonine were detected, a high accumulation of 16-hydroxytabersonine was observed, reaching a similar order of magnitude as 16-methoxytabersonine epoxide. In addition, we noted that this metabolite accumulation remained stable over time, even 48 h after feeding. This suggested that methylation of 16-hydroxytabersonine could represent a concrete bottleneck that needs to be solved to improve vindoline production.

### 2.2. Evaluation of S. cerevisiae Cell Permeability to 16-Hydroxytabersonine

**Figure 4:**
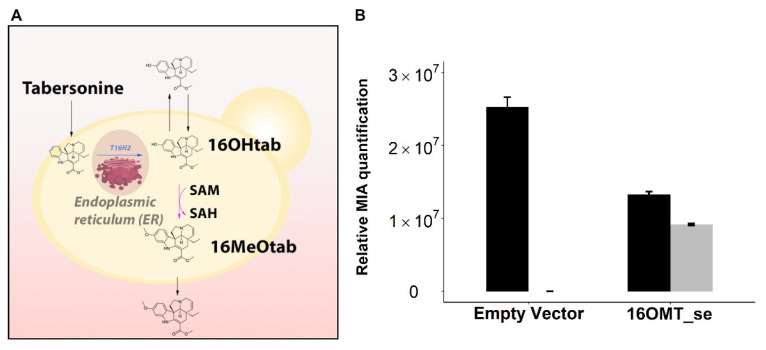
Cell permeability to 16-hydroxytabersonine (16OHtab). (**A**): Illustration of the pathway architecture in the inducible yeast strains from tabersonine to 16-methoxytabersonine (16MeOtab). (**B**): Permeability was evaluated by feeding yeasts (16OMT_se strain: episomal expression of 16OMT (se: single expression), Empty Vector: yeast strain harboring empty episomal plasmid) with 16-hydroxytabersonine. Alkaloids were quantified by UPLC-MS in the yeast culture medium 24 h post-feeding with 16-hydroxytabersonine (250 µM). Black = 16-hydroxytabersonine, grey = 16-methoxytabersonine. Error bars correspond to the standard error of biological replicates (*n* = 3). MIA composition of the yeast culture medium is expressed as relative peak areas.

Since 16-hydroxytabersonine is highly accumulated in yeast culture medium, we next questioned whether such accumulation could result from a low permeability of this compound toward yeast membranes. As previously observed [16], and confirmed in this work, the vindoline biosynthetic intermediates are continuously excreted by yeast in the culture medium and re-internalized, allowing downstream MIA conversion. While T16H2 is anchored to the endoplasmic reticulum (ER, [43]), the methylation catalyzed by *C. roseus* 16OMT takes place in the cytosol and could be drastically reduced if 16-hydroxytabersonine remains in the culture medium ([37], Figure 4A). To test the capacity of 16-hydroxytabersonine import, yeasts were transformed with the *C. roseus* 16OMT-expressing plasmid or the corresponding empty vector (Table 1) and fed with 250 µM of 16-hydroxytabersonine for 24 h before analysis of the MIA content of the culture medium. While only 16-hydroxytabersonine was detected for the control strain transformed with the empty vector, the formation of 16-methoxytabersonine resulting from the methylation of 16-hydroxytabersonine was observed for the yeast strain expressing 16OMT (Figure 4B). 

This result thus showed that 16-hydroxytabersonine, originally fed outside of the cells, is accessible to 16OMT and established that *S. cerevisiae* cells are able to uptake 16-hydroxytabersonine from the culture medium. Consequently, this suggests that the accumulation of 16-hydroxytabersonine is likely to be associated with an insufficient 16OMT activity inside yeast cells.

### 2.3. Optimization of 16OMT Activity in Yeast

#### 2.3.1. Targeting 16OMT to the Endoplasmic Reticulum to Favor Metabolic Channeling

To promote 16OMT activity in yeast cells, we first investigated the effect of enzyme subcellular localization on the MIA biosynthetic flux. Targeting enzymes to specific organelles and enzyme physical associations have been tested previously to improve reaction rate and to avoid biosynthetic intermediate losses [50,51]. For instance, altering the localization of *S. cerevisiae* alcohol acyltransferase from the mitochondria to ER and intracellular lipid droplets enhanced 23-fold and 3-fold its expression and enzymatic activity, respectively [52]. Alternatively, insulating *Papaver somniferum* codeine reductase to the ER significantly increased enzyme specificity and morphine titer in yeast [53]. 

**Figure 5:**
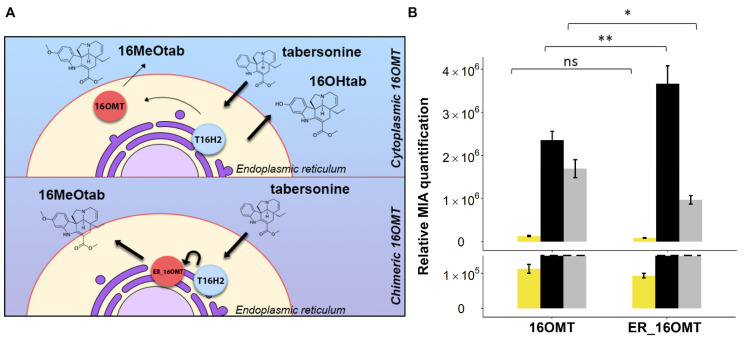
Localization approach for metabolic channeling. (**A**): Schematic illustration of ER-anchored 16OMT (ER_16OMT) construction and its hypothetical metabolic channeling. (**B**): The effect of anchoring 16OMT to ER was evaluated by feeding yeasts (16OMT strain: episomal expression of 2xT16H2 and native 16OMT; ER_16OMT strain: episomal expression of 2xT16H2 and ER_16OMT) with tabersonine. Alkaloids were quantified by UPLC-MS in the yeast culture medium 24 h post-feeding with tabersonine (250 µM). The dashed line represents the scale cut for the visualization of accumulated intermediates of low volume. Statistical analyses were performed with a two-tailed *t*-test (*p* <= 0.1, *: *p* <= 0.05, **: *p* <= 0.01, ns: not significant). Light yellow = tabersonine, black = 16-hydroxytabersonine, grey = 16-methoxytabersonine. Error bars correspond to the standard error of biological replicates (*n* = 3). MIA composition of the yeast culture medium is expressed as relative peak areas.

Given the distinct intracellular localizations of T16H2 and 16OMT in *C. roseus* cells [37], we tried to favor the metabolic channeling between both enzymes by prompting the anchoring of 16OMT to the ER. A chimeric 16OMT bearing the T16H2 transmembrane helix ([43]; Figure S2) was thus created and the resulting ER localization was confirmed in vivo (Figure S3). Coexpressing T16H2 in tandem with the ER-anchored 16OMT (ER_16OMT) was next achieved to potentially facilitate the metabolic channeling between both enzymes (Table 1; Figure 4A). As previously observed, an almost complete consumption of tabersonine was monitored for both strains (Figure 5). However, while 16 methoxytabersonine was produced at a level close to that of 16-hydroxytabersonine in the strain bearing a cytosolic 16OMT, a substantial decrease of the production of this compound was observed with the ER_16OMT. This lowering tended to suggest that ER anchoring altered 16OMT activity as a possible result of a decrease of 16OMT intrinsic activity or 16OMT enzyme amount. This strategy was thus not retained for methoxylation improvement.

#### 2.3.2. Testing a Distinct 16OMT Isoform and Increasing OMT Gene Copy Number to Limit 16-Hydroxytabersonine Accumulation

**Figure 6:**
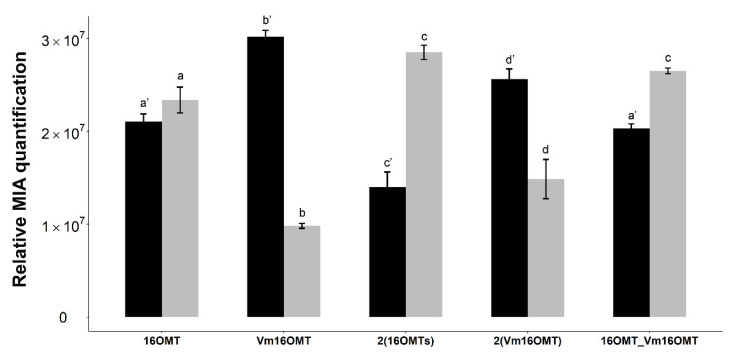
Comparison of 16OMT orthologue activities and effect of gene copy number on tabersonine methoxylation. Yeasts (16OMT strain: episomal expression of 2xT16H2 and 1x16OMT; Vm16OMT strain: episomal expression of 2xT16H2 and 1xVm16OMT; 2(16OMTs): episomal expression of 2xT16H2 and 2x16OMT; 2(Vm16OMT) strain: episomal expression of 2xT16H2 and 2xVm16OMT; 16OMT_Vm16OMT strain: episomal expression of 2xT16H2, 1x16OMT and 1xVm16OMT) were fed with tabersonine (250 µM) before analysis of the MIA content in the culture medium by UPLC-MS. Statistical analyses were performed with ANOVA followed by a Tukey test. Same letters express no significant difference between the means of the same MIA at 5% of significance: **a’**, **b’**, **c’**, **d’** = 16-hydroxytabersonine and **a**, **b**, **c**, **d** = 16-methoxytabersonine. Black = 16-hydroxytabersonine, grey = 16-methoxytabersonine. Error bars correspond to the standard error of biological replicates (*n* = 3). MIA composition of the yeast culture medium is expressed as relative peak areas.

Promoting specialized metabolite synthesis in yeast can be achieved through the selection and expression of the most active orthologues of enzymes displaying low activity. A nice example of this strategy was recently described for phenylpyruvate reductase to optimize the synthesis of tropane alkaloids in heterologous hosts [31,32]. We thus took advantage of the recently published orthologue of 16OMT from *Vinca Minor* (Vm16OMT) and measured its 16-hydroxytabersonine methylation activity in yeast [54]. Vm16OMT was co-expressed using an inducible episomal plasmid along with two copies of T16H2 and compared to the similar strain bearing the *C. roseus* 16OMT, generated previously (Table 1). Again, tabersonine methoxylation was evaluated by measuring the amount of 16-methoxytabersonine in the culture medium. Surprisingly, while Vm16OMT and *C. roseus* 16OMT display similar catalytic properties [54], Vm16OMT seemed to be 4.5 times less active based on 16-methoxytabersonine accumulation (Figure 6). This decreased activity may have resulted from a low expression of Vm16OMT and suggested that this orthologue cannot substitute *C. roseus* 16OMT in our working conditions. While new orthologues can be tested after their identification, *C. roseus* 16OMT remains the best orthologue tested so far.

Obviously, improving a low enzymatic activity in heterologous hosts can also be achieved by adjusting gene copy number as we did with T16H2 (Figure 2). As a consequence, an additional copy of *C. roseus* 16OMT was first expressed along with the two copies of T16H2. Interestingly, in these conditions, the accumulation of 16-hydroxytabersonine decreased down to circa 50% of the amount of the 16-methoxytabersonine accumulated in the culture medium compared to production with one 16OMT copy (Figure 6). Albeit less pronounced, a similar result was also obtained while expressing 2 copies of Vm16OMT versus a single Vm16OMT. Since *O-*methyltransferases operate as dimers, with heterodimers displaying modified kinetic parameters compared to homodimers, *C. roseus* 16OMT was also expressed in tandem with Vm16OMT. However, no improvement of 16-methoxytabersonine production was observed in this condition compared to that of the yeast strain expressing two copies of *C. roseus* 16OMT. Overall, all these results established that increasing 16OMT gene copy number is an efficient strategy to limit 16-hydroxytabersonine accumulation, with *C. roseus* 16OMT being the most active orthologue.

**Figure 7:**
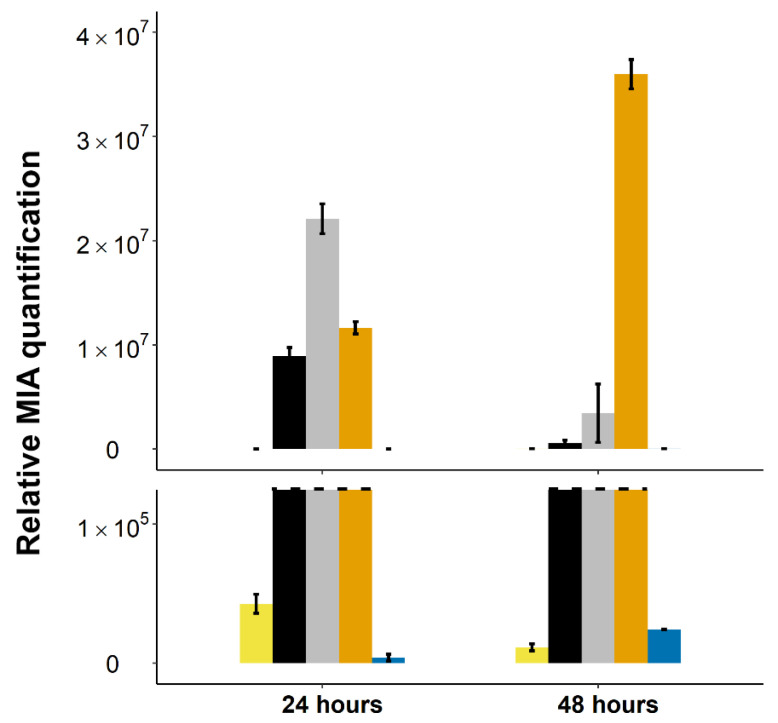
Evolution of MIA biosynthetic intermediates in the medium culture of yeast harboring episomal plasmids containing two copies of T16H2 and *C. roseus* 16OMT and one copy of T3O (2(16OMTs)_T3O strain). Alkaloids were quantified by UPLC-MS in the yeast culture medium 24 h post-feeding with tabersonine (250 µM). The dashed line represents the scale cut for the visualization of low accumulated intermediates. Error bars correspond to the standard error of biological replicates (*n* = 3). Light yellow = tabersonine, black = 16-hydroxytabersonine, grey = 16-methoxytabersonine, dark yellow = 16-methoxytabersonine epoxide, blue = tabersonine epoxide. MIA composition of the yeast culture medium is expressed as relative peak areas.

Based on this observation, we next evaluated the impact of the expression of a second *C. roseus* 16OMT gene copy on the metabolic flux and the production of 16 methoxytabersonine epoxide. The yeast strain co-expressing two copies of both T16H2 and *C. roseus* 16OMT was further transformed to episomally express T3O (Table 1). The MIA content of the culture medium was analyzed 24 and 48 h after tabersonine feeding (Figure 7). In these conditions, the consumption of tabersonine was almost complete with a very low accumulation of tabersonine epoxide, confirming the positive effect of the two T16H2 copies. However, as compared to our original result (Figure 2), a different profile of downstream MIAs was observed at 24 h. We measured a main accumulation of 16-methoxytabersonine, confirming the increased capacity of the engineered yeasts to methylate 16-hydroxytabersonine. It did not result in an increased amount of 16-methoxytabersonine epoxide, thus suggesting that T3O activity could be limiting in this condition. In agreement with this hypothesis, an interesting evolution of the MIA content was monitored at 48 h (Figure 7). Firstly, we noted that 16-hydroxytabersonine was almost fully consumed and the resulting 16-methoxytabersonine followed a similar trend, indicating that both compounds continued to enter the biosynthetic flux. Secondly, we observed that 16-methoxytabersonine epoxide became by far the main accumulated MIA in contrast to our initial observation (Figure 2), suggesting that the conversion of tabersonine was of very high efficiency in these conditions.

These results thus reinforced our previous finding regarding the positive effect of the second *C. roseus* 16OMT on tabersonine conversion. They also shed light on a possible second limiting step catalyzed by T3O that necessitated more time to catalyze the reaction. This is in agreement with the poor T3O catalytic properties reported previously [16,46]. Above all, they confirmed that the optimization of tabersonine methoxylation has a positive effect on 16-methoxytabersonine epoxide biosynthesis.

### 2.4. Stable Integration of the Four First Genes of the Vindoline Pathway Paves the Way for High Vindoline Production

Finally, to improve the limiting activity of T3O compared to the high production of 16-methoxytabersonine, additional modifications of engineered yeast strains were performed. The relative proportion of several episomal plasmids can be highly variable and may affect enzyme populations and their respective activity. To prevent these negative effects, the first four genes of the vindoline pathway were thus stably integrated into the yeast genome. A set of integrative plasmids that allow recombination at classical metabolic markers was thus created along with a yeast strain invalidated for the corresponding metabolic genes, as described in the Methods section. We specifically chose the CEN.PK yeast strain since the potential of this strain is well recognized in metabolic engineering approaches [55]. Two copies of T16H2, *C. roseus* 16OMT and one copy of T3O and *C. roseus* cytochrome P450 reductase (CPR) were first integrated into the yeast genome (Table 1). Confirmation of gene integration in the resulting stable_2(16OMT)s strain was obtained by performing PCR on genomic DNA (Figure S4).

**Figure 8:**
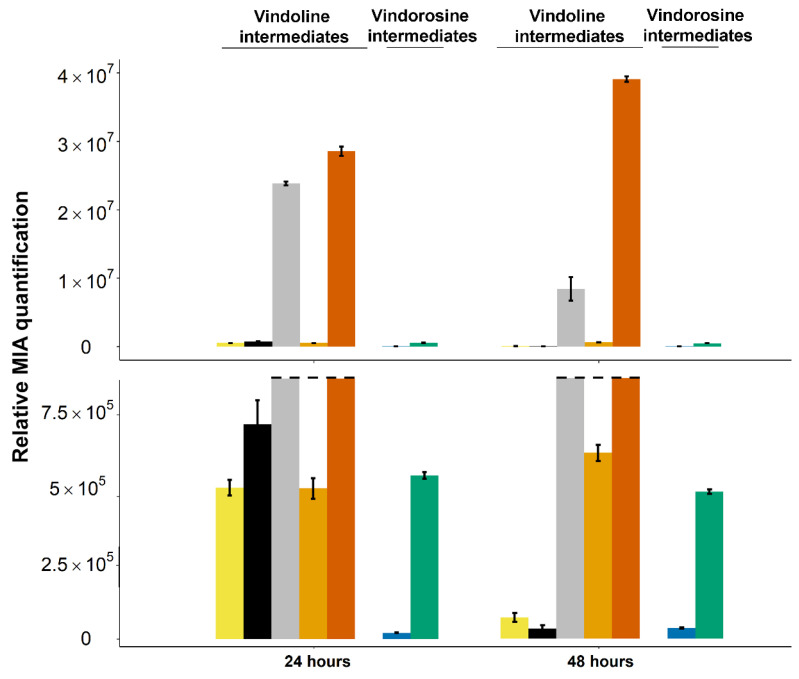
Evolution of MIA biosynthetic intermediates in the culture medium of yeast stably expressing two copies of T16H2 and *C. roseus* 16OMT and one copy of T3O and T3R (Stable_2(16OMT)s). Alkaloids were quantified by UPLC-MS in the yeast culture medium 24 h post-feeding with tabersonine (250 µM). The dashed line represents the scale cut for the visualization of low accumulated intermediates. Light yellow = tabersonine, black = 16-hydroxytabersonine, grey = 16-methoxytabersonine, dark yellow = 16-methoxytabersonine epoxide, orange = 16-methoxy-2,3-dihydro-3-hydroxytabersonine, blue = tabersonine epoxide, green = 2,3-dihydro-3-hydroxytabersonine. Error bars correspond to the standard error of biological replicates (*n* = 3). MIA composition of the yeast culture medium is expressed as relative peak areas.

To limit the production of the undesired tabersonine epoxide, we decided not to increase the T3O gene copy number, which would have negatively imbalanced the competition between T16H2 and T3O for tabersonine access. Instead, we integrated a copy of T3R, the subsequent enzyme of the pathway, to consume the T3O product and potentially increase the biosynthetic flux. In this condition, 24 h after tabersonine feeding, we observed a huge consumption of tabersonine and a limited presence of 16-hydroxytabersonine, confirming that co-expressing two copies of T16H2 and *C. roseus* 16OMT prevents the accumulation of this compound (Figure 8). Only minute quantities of tabersonine epoxide and derivates (2,3-dihydro-3-hydroxytabersonine) were found. In addition, while 16-methoxytabersonine was still accumulated in high amounts, 16-methoxytabersonine epoxide was only detected at trace levels and 16-methoxy-2,3-dihydro-3-hydroxytabersonine became the most abundant MIA produced. Compared to our previous results (Figure 7), this suggested that addition of T3R may promote T3O activity, leading to an increased accumulation of downstream metabolites (Figure 8). This general trend was further confirmed at 48 h since the consumption of the biosynthetic intermediates continued to maximize the production of 16-methoxy-2,3-dihydro-3-hydroxytabersonine, which was 80% of the total produced MIAs (Figure 8; Figure S5).

Together, these results confirmed that the fine-tuning of gene copy number allows us to orient the metabolic flux toward the synthesis of the expected compound. For both yeast strains, stably or episomally expressing the vindoline pathway genes, altering the gene copy number reduced the accumulation of biosynthetic intermediates and avoided the hijacking of tabersonine for the synthesis of vindorosine precursors, thus addressing the first bottlenecks in vindoline precursor production.

## 3. Materials and Methods

### 3.1. Plasmid Construction

The galactose-inducible episomal vectors used in this study were pYeDP60 [56] and pESC vectors series purchased from Agilent (Santa Clara, CA, USA). All the genes cloned in pESC vectors were driven by *GAL10* promoter, except for T3O placed under *GAL1* promoter control (Table 1).

Integrative plasmids with bidirectional promoters were generated using pDONR221, pRS303, or pRS305 backbones. *S. cerevisiae* elements were PCR-amplified (Phusion™ High-Fidelity, ThermoFisher, Waltham, MA, USA) from *S. cerevisiae* gDNA. The promoters were amplified using specific primers containing overlap sequences (forward primers) to further create bidirectional pairs and *Spe*I*/XbaI* restriction sites (reverse primers) (Table S1) for downstream ORF cloning. The obtained DNA fragments were purified (PCR clean-up kit, Machery-Nagel, Düren, Germany) and combined by overlap PCR using promoter reverse primers. The plasmid pURAK (pDONR221 backbone) was constructed by cloning the bidirectional promoter pair of *S. cerevisiae* glycolytic genes *TEF1/TDH3* between *Spe*I and *Xba*I sites, and terminators of the *IDP1* gene between *Sac*I and *Spe*I, and the *PRM5* gene between *Xba*I and *Xho*I. The *URA3* gene was cloned in the *Pvu*II site. The plasmid pHISA (pRS303 backbone) was generated by cloning the bidirectional promoter pair of glycolytic genes *TEF1*/*PGK1* between *Spe*I and *Xba*I sites, and terminators of the *CPS1* gene between *Sac*I and *Spe*I and the *PRM5* gene between *Xba*I and *Xho*I. The plasmid pLEUA (pRS305 backbone) was constructed by cloning the bidirectional promoter pair of glycolytic genes *TEF1*/*PGK1* between *Spe*I and *Xba*I sites and terminators of the *CPS1* gene between *Sac*I and *Spe*I and the *HIS5* gene between *Xba*I and *Xho*I.

The plasmid pJDC1144 was created by cloning the *ARG3* gene in the *Eco*RV site of pDONR221, making a *Nco*I-*Eco*RV deletion in the *ARG3* and finally cloning the *URA3* gene in the *Pvu*II site. The plasmid pJDC256 was created by cloning the *TDH3* gene promoter and the *CYC1* gene terminator in the *Pvu*II site of plasmid pDONR221 and cloning the *C. roseus CPR2* gene optimized for yeast expression by Eurofins (Luxembourg, Luxembourg) in the *Bam*HI-*Eco*RI sites.

The genes of interest were amplified by PCR (Phusion™ High-Fidelity, ThermoFisher) using the primers containing *Spe1*, *BamHI*, *XbaI,* or *Nhe1* restriction sites for downstream ORF cloning (Table S1), followed by restriction enzyme digestion and ligation into the selected plasmids. The cDNA from leaves of *C. roseus* variant ‘Little Bright Eye’ was used as a template. The cDNA was produced from total *C. roseus* RNA following the RevertAid Reverse Transcriptase manufacturer’s instructions (ThermoFisher).

Vector construction and ORF cloning were performed following standard molecular biology methods, which were carried out with *Escherichia coli* TOP10 cells and Luria-Bertani culture medium supplemented with the respective antibiotic for transformant selection (100 µg/mL ampicillin or 50 µg/mL kanamycin).

### 3.2. Yeast Strains

For the galactose-inducible vector system, *S. cerevisiae* WAT11 strain was employed, where the cytochrome P450 reductase gene from *Arabidopsis thaliana* (AtR1) was integrated into the genome [56].

For the integrative vector system, CEN.PK2-1C (MAT*a ura3-52 his3Δ1 leu2-3/112 trp1-289 MAL2-8^c^ SUC2*) strain (Euroscarf, Oberursel, Germany) was transformed by *Bgl*II-linearized pJDC144 to generate the *arg3* mutant, creating the strain JDC058 after excision of the plasmid by selecting yeast clones auxotrophic for uracil and arginine. This strain was further transformed by pJDC256, which carries the yeast optimized *C. roseus CPR* open reading frame (ORF) under the control of the *TDH3* gene promoter linearized by *Nco*I for the insertion at the *ARG3* locus (JDC058_CPR strain). The strains generated downstream are listed in Table 1.

### 3.3. Yeast Transformations and Culture

For the generation of *S. cerevisiae* WAT11 inducible strains, yeast competent cells were prepared prior to transformation [57]. The competent cells were transformed by electroporation with expression plasmids according to Table 1. The yeast selection was done by synthetic complete drop-out (SC) plates containing 0.67% yeast nitrogen base, 2% agar, 2% dextrose, and 0.05% DOB according to vector markers. Yeast overnight pre-cultures were performed in drop-out selection liquid medium for 16 h followed by the induction in YPGal medium (1% bactopeptone, 1% yeast extract, and 2% Gal) for 5 h at 28 °C with constant agitation of 200 rpm prior to the feeding with 250 µM of tabersonine (ChromaDex, Los Angeles, CA, USA) or 16-hydroxytabersonine as described by [43].

To generate *S. cerevisiae* CEN.PK stable strains, the previously constructed JDC058-CPR strain was transformed following the LiAc/PEG method [58] with the linearized (*StuI* for pURAK, *NheI* for pHISA, and *EcoRV* for pLEUA) integrative plasmids (Table 1). Selection of transformants was performed on synthetic complete drop-out (SC) plates according to the auxotrophic markers. Stable strains were further inoculated in liquid yeast extract-peptone-dextrose medium (YPD, 10 g L^−1^ of yeast extract, 20 g L^−1^ of peptone and 20 g L^−1^ of glucose) for 16 h under constant agitation of 200 rpm, followed by the 20-fold dilution in fresh YPD medium and feeding with 250 µM of tabersonine (ChromaDex) or 16-hydroxytabersonine [43] in the final volume of 200 µL.

Gene integration in the stable_2(16OMT)s yeast strain was confirmed by PCR amplifications performed on yeast genomic DNA extracted with the kit Plant II (Macherey-Nagel) following the manufacturer’s instructions. The wild type CEN.PK genomic DNA was also extracted to be used as a control. PCR reactions (Phusion™ High-Fidelity, ThermoFisher) were conducted using both DNAs as template and primers, as described in Table S1, before analysis in 1% agarose gels.

### 3.4. Construction of the Chimeric 16OMT

Prior to the construction of chimeric 16OMT, the transmembrane helix was amplified by PCR (Phusion™ High-Fidelity, ThermoFisher) using T16H2helix_FOR and T16H2helix_REV primers (Table S1) and pYEDP60_T16H2 plasmid as a template. The obtained fragment was further fused with the amplification product of 16OMT by PCR using the T16H2helix_FOR and the internal primer helix_OMT_REV (Table S1).

### 3.5. Yeast Metabolite Analysis

The bioconversion assays were followed by the separation of yeast extracellular media via centrifugation (8000 *g* for 10 min) and 18-fold dilution of supernatant in 100% methanol. The diluted samples were analyzed by UPLC-MS after 15 min of centrifugation at 16,000× *g*. The alkaloids were identified in the selected ion-monitoring mode as described in [59] (Figure S1). The results were processed using the QuanLynx™ software (Waters, Milford, MA, USA) in peak areas (ions count).

### 3.6. Subcellular Localization in C. roseus Cells

The subcellular localization of the chimeric 16OMT was confirmed by cloning the fusion PCR product into pSC-A plasmids. The cloning was performed in *SpeI* restriction sites in order to position YFP at the C-terminus of the chimeric protein while the ER-helix peptide signal was located at the N-terminus. The generated vector was further used for transient transformation of *C. roseus* cells by particle bombardment, and the images were prepared according to [37]. The vectors were co-transformed with plasmids expressing the CFP-ER (CD3-954) from the ABRC (http://www.arabidopsis.org, accessed on 10 June 2021) or CFP-nucleocytoplasm obtained from [37].

## 4. Conclusions

While the supply of vinblastine and vincristine suffers from recurrent shortages [60], there is an urgent need to develop alternative production approaches to maintain a continuous access to these essential drugs. Using yeast cell factories to produce the precursors of these compounds is one of the promising alternatives. A first proof-of-concept of vindoline production in yeast was described through precursor directed synthesis [16]. However, the low vindoline biosynthesis and the hijacking of the precursor tabersonine towards the production of the unwanted vindorosine indicated that further optimizations were required. In this context, we have shown here that tabersonine methoxylation constitutes a real bottleneck in vindoline precursor synthesis due to (i) the competition between tabersonine hydroxylation and epoxidation and (ii) the accumulation of 16-hydroxtabersonine over time. By adjusting the copy number of the first two genes of the vindoline biosynthetic pathway, namely T16H2 and *C. roseus* 16OMT, we successfully addressed these two problems and created a yeast strain producing the fourth biosynthetic intermediate of vindoline without massive accumulation of other intermediates or undesired side-products. This optimization of tabersonine methoxylation will probably pave the way towards the future development of yeast cell factories producing vindoline at an industrial scale. In addition, it could also guide the heterologous reconstitution of other biosynthetic pathways in yeast involving two successive steps of hydroxylation and methylation. However, after this first optimization, the balance of gene copy will probably need to be readjusted in the context of the full vindoline pathway reconstitution since new bottlenecks resulting from increased synthesis of early precursors may appears later in the pathway. Future studies will have to address this concerted gene expression at the whole pathway level and improve the intrinsic activity of the last enzymes of the pathway. Such an overall increase of enzyme activity will probably involve the expression of additional proteins involved in the regeneration of some MIA pathway enzyme cofactors such as NADPH [22]. Delocalization of MIA biosynthetic enzymes to new subcellular compartments such as vacuoles or peroxisomes may also represent an interesting option to maximize the metabolic flux or avoid the accumulation of undesired/toxic intermediates [31,51,61]. Finally, controlled fed-batch fermentations of the newly developed yeast strains in bioreactors will probably increase MIA synthesis up to industrial scales.

## Figures and Tables

**Table 1:** Yeast strains and the respective plasmids used for the transformation.

Strains	Plasmids
***Inducible WAT11 strains***	
T16H2+T3O	pYEDP60_T16H2, pESC-TRP_empty, pESC-HIS_empty, pESC-LEU_T3O
2(T16H2)+T3O	pYEDP60_T16H2, pESC-TRP_T16H2, pESC-HIS_empty, pESC-LEU_T3O
2(T16H2)_T3O	pYEDP60_T16H2, pESC-TRP_T16H2, pESC-HIS_empty, pESC-LEU_16OMT_T3O
16OMT_se *	pESC_LEU_16OMT
ER_16OMT	pYEDP60_T16H2, pESC-TRP_T16H2, pESC-HIS_empty, pESC-LEU_EROMT
16OMT	pYEDP60_T16H2, pESC-TRP_T16H2, pESC-HIS_16OMT, pESC-LEU_empty
2(16OMTs)	pYEDP60_T16H2, pESC-TRP_T16H2, pESC-HIS_16OMT, pESC-LEU_16OMT
Vm16OMT	pYEDP60_T16H2, pESC-TRP_T16H2, pESC-HIS_Vm16OMT, pESC-LEU_empty
2(Vm16OMT)	pYEDP60_T16H2, pESC-TRP_T16H2, pESC-HIS_Vm16OMT, pESC-LEU_Vm16OMT
16OMT_Vm16OMT	pYEDP60_T16H2, pESC-TRP_T16H2, pESC-HIS_16OMT, pESC-LEU_Vm16OMT
T16H	pYEDP60_T16H2, pESC-TRP_T16H2, pESC-HIS empty, pESC-LEU_empty
16OMT_T3O	pYEDP60_T16H2, pESC-TRP_T16H2, pESC-HIS empty, pESC-LEU_16OMT_T3O
2(16OMTs)_T3O	pYEDP60_T16H2, pESC-TRP_T16H2, pESC-HIS_16OMT, pESC-LEU_16OMT_T3O
Empty_vectors	pYEDP60_empty, pESC-TRP_empty, pESC-HIS_empty, pESC-LEU_empty
***Stable JDC058_CPR strains***	
stable_2(16OMT)s	pHISA_pGK1-T16H2-tPRM5_pTEF1-16OMT-tCPS1, pURAK_pTDH3-T16H2-tPRM5_pTEF1T3O-tIDP1,pLEUA_pTEF1-16OMT-tCPS1_pGK1-T3R-tHIS5

* 16OMT_se: 16OMT single expression.

## Data Availability

Data is contained within the article.

## Supplementary Materials

The following are available online, Table S1: List of primers, Figure S1: UPLC-MS/MS chromatograms of the natural products produced by the yeast strains. Figure S2: Fusion of the T16H2 transmembrane helix to the N-terminal end of 16OMT (ER_16OMT). Alignment of the first 55 residues of T16H2 with ER_16OMT. The red rectangle highlights the added sequence including the predicted transmembrane helix of identified in T16H2. Figure S3: Subcellular localization of 16OMT and EROMT in C. roseus cells. Cells were transformed transiently with 16OMT-YFP (A–D) and EROMT-YFP (E-H) expressing vectors in combination with CFP-nucleocytosolic or CFP-ER marker (second column). Co-localization of the two fluorescence signals appeared in the merged image (C, G). The morphology is observed with differential interference contrast (DIC). Bar: 10 um. Figure S4: Phusion PCR amplification of the integrated genes from the vindoline’s pathway using genomic DNA from the designed stable yeast (Stable_2(16OMT)s) and genomic DNA from wild type CEN.PK (CEN.PK WT). The yeast gene SAM2 (S-adenosylmethionine synthetase) was used as positive control. T16H2: tabersonyne-16-hydroxylase, 16OMT: tabersonine-16-O-methyltransferase, T3O: tabersonine 3-oxygenase, T3R: tabersonine 3-reductase, CPR: optimized C. roseus CPR, SAM2: S-adenosylmethionine synthetase. Figure S5: Evolution of the accumulation of vindoline and vindorosine biosynthetic intermediates in the stable_2(16OMT)s yeast strain fed with tabersonine. Alkaloids were quantified by UPLC-MS in the yeast culture medium before and 24 and 48 hours post-feeding with tabersonine (250 µM). Error bars correspond to the standard error of biological replicates (*n* = 3).

## Sample Availability

Samples of the compounds (16-hydroxytabersonine, 16-methoxytabersonine) are available from the authors.
