# Supplementary material for: Optimization of Tabersonine Methoxylation to Increase Vindoline Precursor Synthesis in Yeast Cell Factories
Source: Molecules. 2021 Jun 11;26(12):3596. doi: 10.3390/molecules26123596 (PMC8231165; doi:10.3390/molecules26123596)
Supplement: Supplementary file 1 [file molecules-26-03596-s001.zip › molecules-1204832-supplementary.pdf]

Supplementary data:

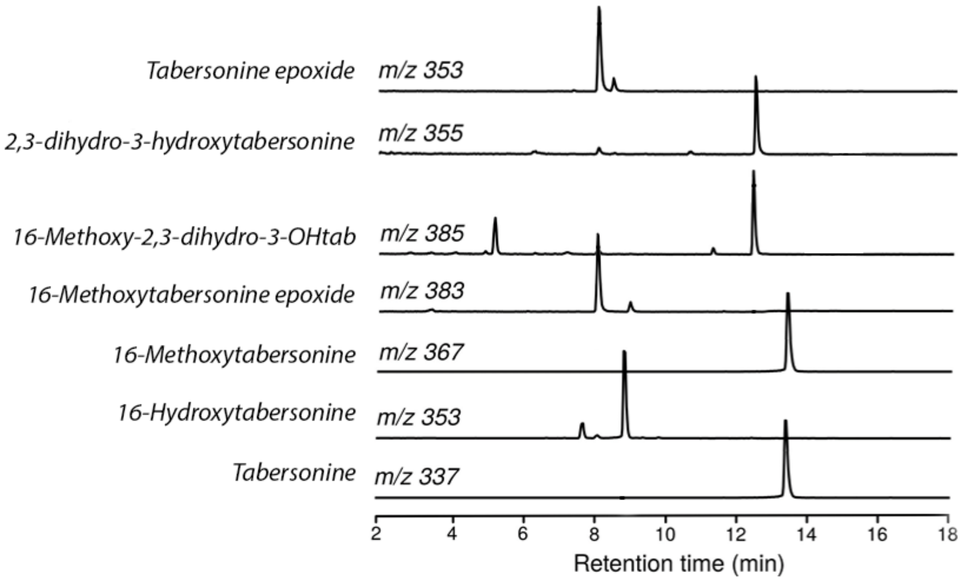

Figure 1. UPLC-MS chromatograms of the natural products produced by the yeast strains.

Table S1: Primers list

Gene	Forward primer	Reverse primer
T16H2	CTGAGAACTAGTTCGGTCATGGAGTTGTATTATTTTTCCACC	CTGAGAACTAGTCTAATATTTACCTTTGAGAGAAGAAGC
16OMT	CTGAGAACTAGTTCGGTCATGGATGTTCAATCTGAGGAGT	CTGAGAACTAGTTCAAGGATAAACCTCAATGAGAC
T3O	CTGAGAACTAGTTCGGTCATGGAGTTTCATGAATCTTCTCCC	CTGAGAACTAGTTCATGCATAGGACGTAGCGA
T3R	CTGAGATCTAGATCGGTCATGGCAATGGCTTCAAAGTC	CTGAGATCTAGATTAGGGTGATTTGAAAGTGTTTCC
T16H2_helix	CTGAGAACTAGTATGGAGTTGTATTATTTTTCCACCTTTG	GCCAGATTTCTTTAGAGTTTTGGCTAA
EROMT (fusion primer)	ACTCTAAAGAAATCTGGCATGGATGTTCAATCTGAGGAGTTCC	CTGAGAACTAGTTCAAGGATAAACCTCAATGAGACTCC
pTEF1	CCAGTCGATTATCATGTTCGCTACCACACACCATAGCTTCAAAATG	CTGAGAACTAGTTTTGTAATTAAAACTTAGATTAGATTGCTATGC
pPGK1	TAGCGAACATGATAATCGACTGGAGACGCGAATTTTTCGAAGAA	CTGAGAACTAGTTCTAGATGTTTTATATTTGTTGTAAAAAGTAGATAATTAC
pTDH3	TAGCGAACATGATAATCGACTGGCTATTTTCGAGGACCTTGTCACCTT	CTGAGAACTAGTTCTAGATTTGTTTGTTTATGTGTGTTTATTCGAAAC

pACT1	CCAGTCGATTATCATGTTCGCTAGTGAAGATGTGGCTGC AAGATT	CTGAGATCTAGAGCGGCCGCGTTAATTCAGTAAA TTTTCGATCTTGG
CPR *C. roseus*	GGATCCCCCTCGAGTTAATTAACGTAATGGATTCTTCCT CAGAAAAGTTAAGC	GAATTCGCTAGCTCTAGAGCGATCGCCCACTTAC CAAACATCACGTAAGTATCTACC
ARG3	CATTACGCTCCTTCGTATTAC	GTGAAATTGGACAACTCGAAGG
tCPS1	GAGCTCTTTAGTCATTGTATGGTC	ACTAGTGCGCAATGATTGAATAGTCAAAG
HIS5	TCTAGAATAGATTAATTTAAACAGTATATGTACAG	GTCGACTGATATTTAGCTATATGTACGTTGTTAG
URA3	GATTCGGTAATCTCCGAAC	TTTGTGAGTTTAGTATACATGC
IDP1	GAGCTCCAACAATAAGGTATATATATTTATGATAACAG	ACTAGTTCGAATTTACGTAGCCCAATC
PRM5	TCTAGAAAACTTTTATGATATTTTGCAATATTTTTTTTAA GC	CTCGAGTATAATAAGACACGGACGCAC
SAM2	CTGAGAACTAGTATGTCCAAGAGCAAAACTTTCTTATTT ACC	CTGAGAACTAGTTTAAAATTCCAATTTCTTTGGTT TTTCC

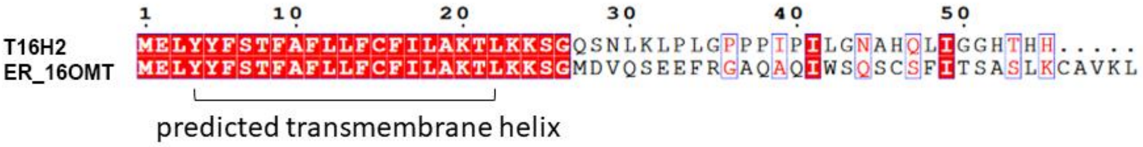

Figure S2: Fusion of the T16H2 transmembrane helix to the N-terminal end of 16OMT (ER_16OMT). Alignment of the first 55 residues of T16H2 with ER_16OMT. The red rectangle highlights the added sequence including the predicted transmembrane helix of identified in T16H2.

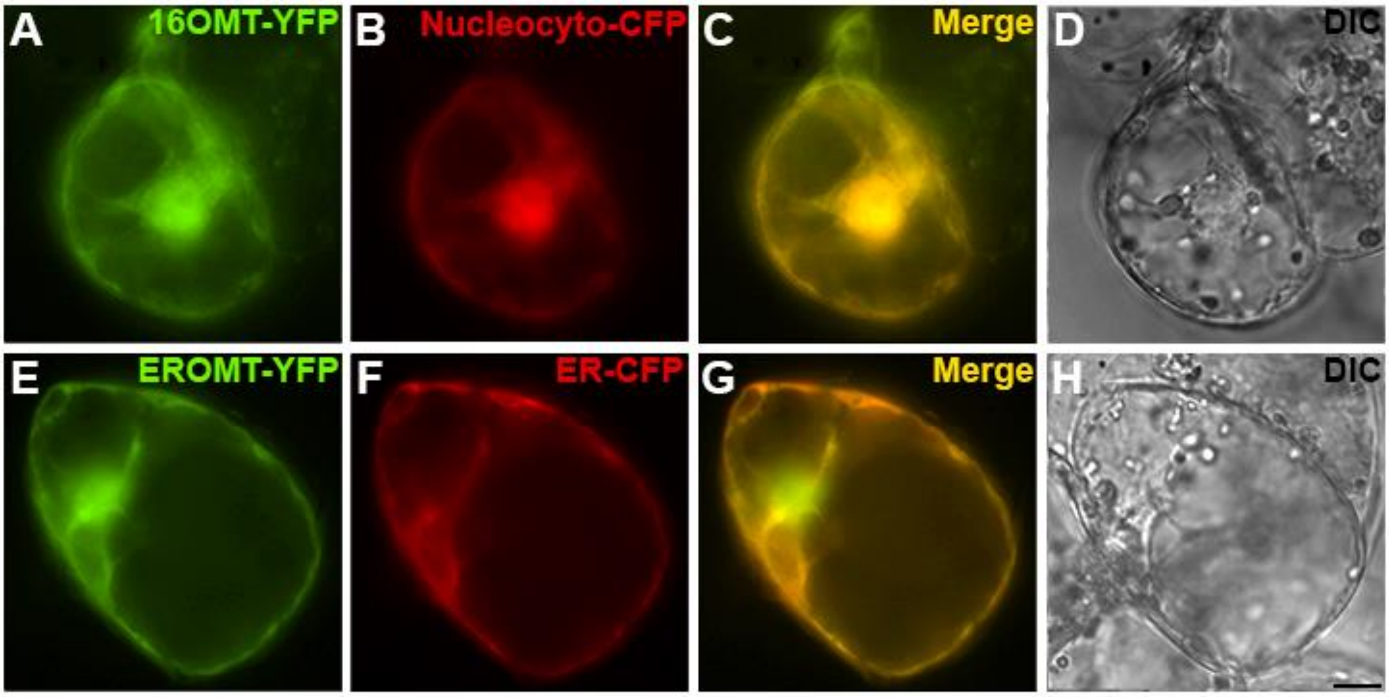

Figure S3: Subcellular localization of 16OMT and EROMT in *C. roseus* cells. Cells were transformed transiently with 16OMT-YFP (A–D) and EROMT-YFP (E-H) expressing vectors in combination with CFP-nucleocytosolic or CFP-ER marker (second column). Co-localization of the two fluorescence signals appeared in the merged image (C, G). The morphology is observed with differential interference contrast (DIC). Bar: 10 um.

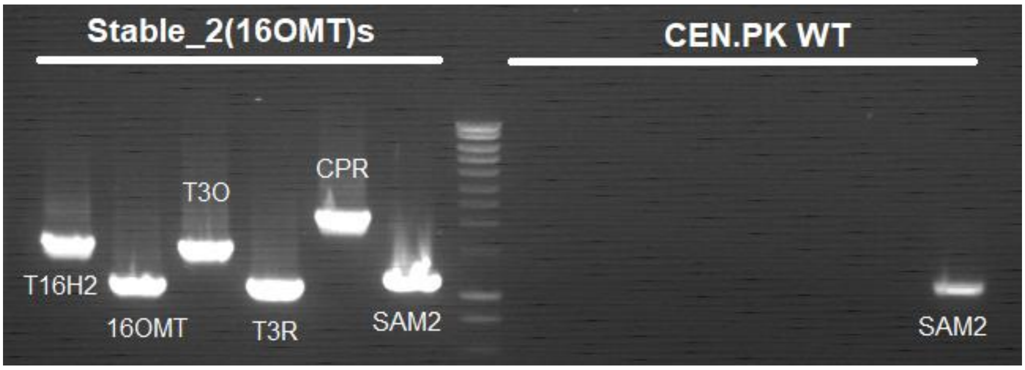

Figure S4: Phusion PCR amplification of the integrated genes from the vindoline's pathway using genomic DNA from the designed stable yeast (Stable_2(16OMT)s) and genomic DNA from wild type CEN.PK (CEN.PK WT). The yeast gene SAM2 (S-adenosylmethionine synthetase) was used as positive control. T16H2: tabersonyne-16-hydroxylase, 16OMT: tabersonine-16-*O*-methyltransferase, T3O: tabersonine 3-oxygenase, T3R: tabersonine 3-reductase, CPR: optimized *C. roseus CPR*, SAM2: S-adenosylmethionine synthetase.

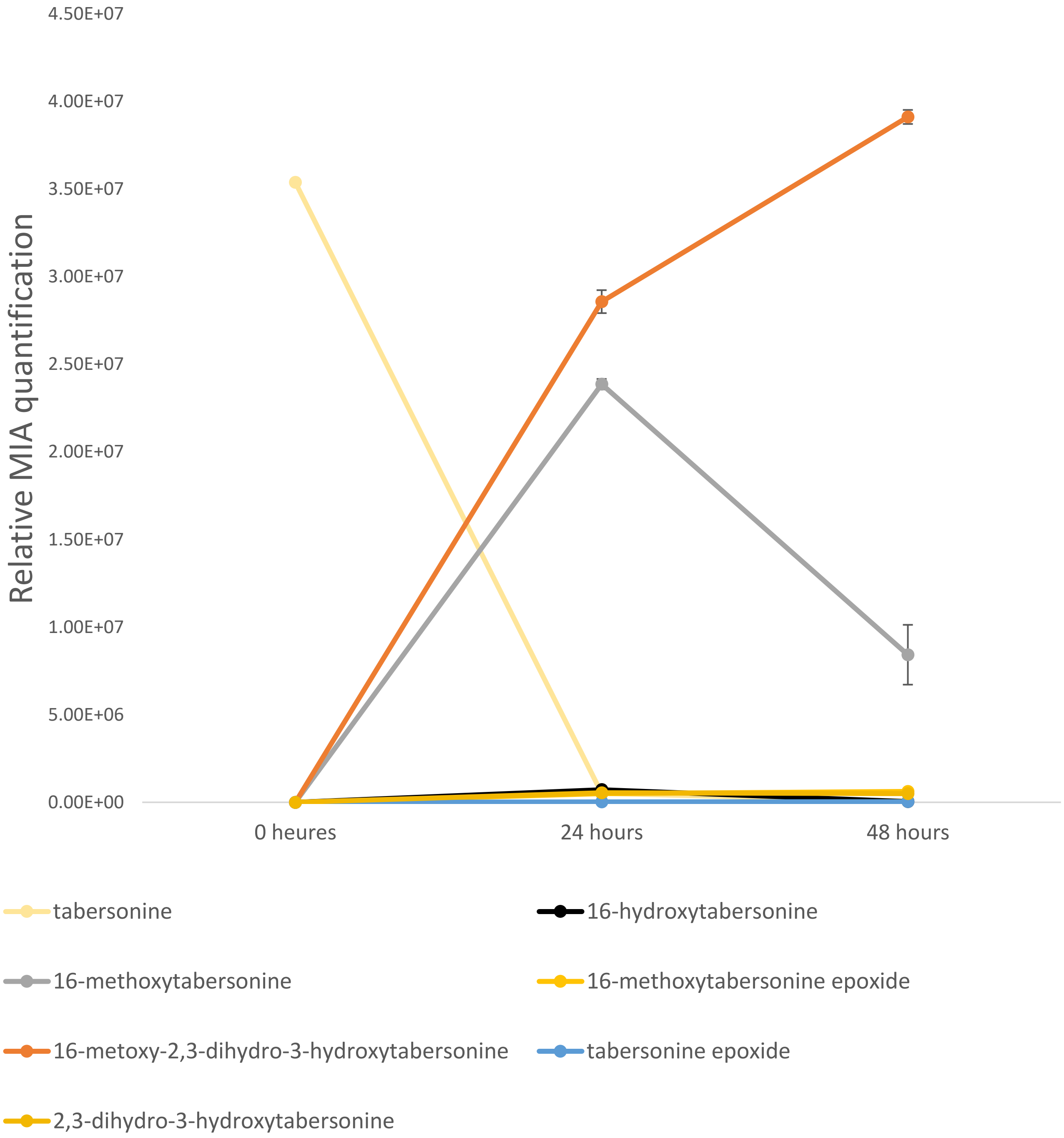

Figure S5: Evolution of the accumulation of vindoline and vindorosine biosynthetic intermediates in the stable_2(16OMT)s yeast strain fed with tabersonine. Alkaloids were quantified by UPLC-MS in the yeast culture medium before and 24 and 48 hours post-feeding with tabersonine (250 µM). Error bars correspond to the standard error of biological replicates (n = 3).
